# Independent Associations of Tumor Necrosis Factor-Alpha and Interleukin-1 Beta With Radiographic Emphysema in People Living With HIV

**DOI:** 10.3389/fimmu.2021.668113

**Published:** 2021-04-14

**Authors:** Rebekka F. Thudium, Hedda Ringheim, Andreas Ronit, Hedda Hoel, Thomas Benfield, Amanda Mocroft, Jan Gerstoft, Marius Trøseid, Álvaro H. Borges, Sisse R. Ostrowski, Jørgen Vestbo, Susanne D. Nielsen

**Affiliations:** ^1^ Viro-immunology Research Unit, Department of Infectious Diseases, Rigshospitalet, University of Copenhagen, Copenhagen, Denmark; ^2^ Department of Infectious Diseases, Copenhagen University Hospital - Amager and Hvidovre Hospital, Hvidovre, Denmark; ^3^ Research Institute of Internal Medicine, Oslo University Hospital Rikshospitalet, Oslo, Norway; ^4^ Department of Clinical Medicine, University of Copenhagen, Copenhagen, Denmark; ^5^ Centre for Clinical Research, Epidemiology, Modelling and Evaluation (CREME), Institute for Global Health, University College London, London, United Kingdom; ^6^ Centre for Health and Infectious Diseases (CHIP), Department of Infectious Diseases, Section 2100, Rigshospitalet, University of Copenhagen, Copenhagen, Denmark; ^7^ Department of Infectious Diseases Immunology, Statens Serum Institut, Copenhagen, Denmark; ^8^ Department of Clinical Immunology, Rigshospitalet, University of Copenhagen, Copenhagen, Denmark; ^9^ Division of Infection, Immunity and Respiratory Medicine, University of Manchester and Manchester University NHS Foundation Trust, Manchester, United Kingdom

**Keywords:** HIV, emphysema, inflammation, non-AIDS comorbidity, interleukin-1 beta, cytokines

## Abstract

**Background:**

People living with HIV (PLWH) have increased systemic inflammation, and inflammation has been suggested to contribute to the pathogenesis of emphysema. We investigated whether elevated cytokine concentrations (interleukin (IL)-1β, IL-1 receptor antagonist (IL-1RA), IL-2, IL-4, IL-6, IL-10, IL-17A, tumor necrosis factor-alpha (TNFα), interferon-gamma (IFNγ), soluble CD14 (sCD14) and sCD163 were independently associated with radiographic emphysema in PLWH.

**Methods:**

We included PLWH from the Copenhagen Comorbidity in HIV Infection (COCOMO) Study without hepatitis B and C co-infection and with a plasma sample and a chest computed tomography scan available. Emphysema plus trace emphysema was defined as the percentage of low attenuation area under −950 Houndsfield Unit (%LAA-950) using a cut-off at 5%. Cytokine concentrations were measured by ELISA or Luminex immunoassays. An elevated cytokine concentration was defined as above the 75^th^ percentile.

**Results:**

Of 783 PLWH, 147 (18.8%) had emphysema. PLWH were predominantly male (86.0%) and 743 (94.9%) had undetectable viral replication. PLWH with emphysema had higher concentrations of TNFα (median (IQR): 8.2 (6.4-9.8) versus 7.1 (5.7-8.6) pg/ml, p<0.001), IL-1β (0.21 (0.1-0.4) versus 0.17 (0.1-0.3) pg/ml, p=0.004) and IL-6 (3.6 (2.6-4.9) versus 3.1 (2.0-4.3) pg/ml, p=0.023) than PLWH without. In a logistic regression model adjusted for age, sex, ethnicity, smoking status, BMI and CD4 nadir, elevated TNFα (adjusted odds ratio (aOR): 1.78 [95%CI: 1.14-2.76], p=0.011) and IL-1β (aOR: 1.81 [95%CI: 1.16-2.81], p=0.009) were independently associated with emphysema. The association between IL-1β and emphysema was modified by smoking (p-interaction=0.020) with a more pronounced association in never-smokers (aOR: 4.53 [95%CI: 2.05-9.98], p<0.001).

**Conclusion:**

Two markers of systemic inflammation, TNFα and IL-1β, were independently associated with emphysema in PLWH and may contribute to the pathogenesis of emphysema. Importantly, the effect of IL-1β seems to be mediated through pathways that are independent of excessive smoking.

**Clinical Trial Registration:**

clinicaltrials.gov, identifier NCT02382822.

## Introduction

During the current combination antiretroviral therapy (cART) era, comorbidities such as chronic pulmonary diseases are more prevalent in people living with HIV (PLWH) than in the background population (1–3). Emphysema, characterized by enlargement of terminal air sacs and destruction of alveolar walls, has been suggested to occur more frequently and at younger age in PLWH than in uninfected controls (4–7). We previously studied radiographic emphysema in PLWH, and although we did not find HIV to be independently associated with emphysema, we found respiratory symptoms to be more prevalent in PLWH with emphysema compared to controls, suggesting a greater clinical impact of emphysema in this population (8). Known risk factors for the development of emphysema include smoking and alpha-1 antitrypsin (AAT) deficiency, but evidence suggest that inflammation may also play a role in the pathogenesis of emphysema (9–11).

Chronic systemic inflammation and immune activation are considered hall-marks of HIV infection, even among those receiving suppressive cART (12). Further, markers of systemic inflammation, such as the pro-inflammatory cytokine interleukin-6 (IL-6), have been tightly linked to comorbidities and mortality in PLWH (13–15). However, the association between inflammatory markers and emphysema in well-treated PLWH has received little attention and is not well described.

In the present study we hypothesized that pro-inflammatory markers of systemic inflammation are independently associated with radiographic emphysema. This hypothesis was explored in a large cohort of mainly well-treated PLWH without hepatitis B and C co-infection.

## Materials and MethodS

### Study Design and Population

In this cross-sectional study, we included PLWH enrolled in the Copenhagen Comorbidity in HIV Infection (COCOMO) study. The COCOMO study (n=1099) is a non-interventional, longitudinal study that was initiated in March 2015 (16). The primary aim of the study is to investigate the burden and pathogenesis of non-AIDS comorbidities in PLWH from the greater Copenhagen area. In the current study we included participants in the COCOMO study with a plasma blood sample and a computed tomography (CT) scan of the chest available and with no evidence of hepatitis B and/or hepatitis C co-infection. A flowchart summarizing the inclusion process is available in the supplement (**Supplementary Figure 1**). Data collection for all participants was performed between March 2015 until November 2016. The COCOMO study was approved by the Committee on Health Research Ethics of the Capital Region of Denmark (approval number: H-8-2014-004) and the Danish Data Protection Agency. Written informed consent was obtained from all participants.

### Data Collection

At baseline, participants underwent physical examination including lung function test by spirometry and height and weight measurements. Body mass index (BMI) calculations and categorization were done according to WHO criteria (17). Blood was drawn for biochemical analyses and plasma was stored in a biobank with subsequent analyses of inflammatory markers. In addition, each participant completed a comprehensive questionnaire regarding lifestyle and health (16). Smoking status was categorized into current, former and never smoking. HIV related variables, including CD4 and CD8 T-cell counts and viral load, were obtained from medical records. CD4/CD8 ratio was stratified into three groups (<0.4, 0.4-1.0, >1.0; low, middle, normal) as done in prior studies (18, 19).

### Biochemical Analyses of Inflammatory Markers

Plasma concentrations of pro- and anti-inflammatory cytokines (pg/ml) were measured using magnetic multiplex assay kits from R&D systems (Minneapolis, Minnesota, USA) as previously described (20). The multiplex assay kits consisted of interleukin-1 beta (IL-1β), IL-2, IL-4, IL-6, IL-10, IL-17A, interferon-gamma (IFNγ) and tumor necrosis factor-alpha (TNFα). Other inflammatory markers, including interleukin-1 receptor antagonist (IL-1RA) (pg/ml), and markers of monocyte activation, sCD14 and sCD163 (ng/ml), were measured in plasma using ELISA kits (R&D systems, Minneapolis, Minnesota, USA).

### Computed Tomography Scanning Procedure

In addition to the physical exam, participants were offered a CT scan of the chest. All CT scans were performed on an Aquilion One Vision Edition scanner (Toshiba Medical Systems, Otawara-shi, Japan) at the Department of Radiology, Rigshospitalet, Copenhagen, Denmark. The following settings were used for image acquisition: 120 kVp, automated exposure control (SD15) and reconstruction with filtered back projection and a soft tissue kernel (1mm slice thickness and 1mm increment). Scans were obtained during a deep inspiratory breath-hold and included the entire lungs. The entire protocol used for spiral image acquisition in chest CT scans has been described in details elsewhere (16).

### Definition of Emphysema

Lung emphysema was assessed quantitatively by densitometry using %LAA-950 which is defined as percentage of lung parenchyma with low attenuation area under a threshold of -950 Hounsfield units (HU) (21). Our primary outcome was emphysema defined as %LAA-950 > 5%, allowing a wide range of emphysematous changes to be included in the study (22). Images from all CT examinations were scored using a specialized lung density program (Vitrea Vital Images, Minnetonka, MN, USA).

For sensitivity analyses, we assessed emphysema according to a semi-quantitative visual score ranging from 0-5: 0, no emphysema (0%); 1, trace emphysema (1-10%); 2, mild emphysema (11-25%), 3, moderate emphysema (26-50%); 4, severe emphysema (51-75%); 5, very severe emphysema (>75%). A single board-certified radiologist scored all scans blinded to the results of the quantitative CT analyses. Visual emphysema was defined as a visual score ≥ 2, as done previously (8).

### Statistical Analyses

Differences in clinical characteristics and plasma concentration of inflammatory markers between PLWH with and without emphysema were assessed using Student´s t-test or Wilcoxon’s rank-sum test for continuous data and Pearson’s χ^2^-test for categorical data, as appropriate. All inflammatory markers were dichotomized using the 3^rd^ quartile as cut-off. Thus, an elevated cytokine concentration was defined as above the 75^th^ percentile. Lung emphysema was regarded as a dichotomous variable (yes/no) and defined as percentage of low attenuation area under -950 Hounsfield units (%LAA-950) > 5% in chest CT scans. Multivariable logistic regression analyses were performed to investigate the association between emphysema and low CD4/CD8 ratio as well as cytokine concentrations, one cytokine at the time. We used two pre-defined models in our analyses; a base model adjusted for age and sex, and a fully adjusted model including potential confounders such as age, sex, ethnicity, smoking status, BMI category and CD4 nadir (6, 10, 23). We then assessed correlation between the inflammatory markers that significantly associated with emphysema using the Spearman method. A final model, potentially including several inflammatory markers was made, ensuring that cytokines with high collinearity was not put in the model together. Finally, we tested for statistical interaction between elevated cytokines and smoking on their effect on the odds for emphysema using logistic regression analyses and the final fully adjusted model listed above. In sensitivity analyses, we tested the inflammatory markers significantly associated with emphysema in primary analyses, in multivariable logistic regression models using the secondary outcome definition (visual emphysema) as the dependent variable. Also, based on previous reports from the literature, explorative analyses on associations between CD4/CD8 ratio and visual emphysema were performed. No adjustments for multiple comparisons were conducted. Statistical analyses were performed using R software version 3.6.1 (24).

## Results

Of 783 PLWH included in the study, 147 (18.8%) had emphysema. The majority of PLWH were males (86.0%) with undetectable viral replication (94.9%) and a mean (SD) age of 51.0 (11.3) years. PLWH with emphysema were older and more likely to be male than PLWH without emphysema (**Table 1**). In addition, PLWH with emphysema had a lower proportion of current smokers, a lower BMI and a higher prevalence of airflow limitation than PLWH without emphysema. There were no differences in cART coverage, current CD4 T-cell count or viral suppression between PLWH with and without emphysema. However, we found a higher proportion of low CD4 nadir (<200 cells per µl) and a longer duration of HIV infection in PLWH with emphysema compared to PLWH without (**Table 1**).

**Table 1:** Clinical characteristics of people living with HIV with and without emphysema.

	Emphysema (n=147)	No emphysema (n=618)	p-value
Age, mean (SD)	57.1 (11.5)	49.7 (10.7)	<0.001
Sex (male), n (%)	141 (95.9)	514 (83.2)	<0.001
Origin			
• Scandinavia, n (%)	121 (82.3)	435 (70.4)	0.299
• Other European, n (%)	13 (8.8)	76 (12.3)	
• Other, n (%)	12 (8.2)	99 (16.0)	
Smoking status			
• Current smokers, n (%)	26 (17.9)	188 (31.1)	<0.001
• Former smokers, n (%)	76 (52.4)	205 (33.9)	
• Never smokers, n (%)	43 (29.7)	212 (35.0)	
BMI, mean (SD)	23.8 (3.2)	25.1 (3.7)	<0.001
BMI category, n (%)			
• Underweight	7 (4.8)	13 (2.1)	0.007
• Normal	91 (61.9)	324 (52.7)	
• Overweight	47 (32.0)	220 (35.8)	
• Obese	2 (1.4)	58 (9.4)	
**Pulmonary function**			
FEV_1_ (L), mean (SD)	3.3 (0.9)	3.5 (0.8)	0.065
FVC (L), mean (SD)	4.6 (1.0)	4.4 (1.0)	0.121
Airflow limitation, LLN, n (%)	30 (20.7)	43 (7.0)	<0.001
AAT deficiency, n (%)	10 (7.1)	27 (4.6)	0.291
**HIV related variables**			
Current CD4 count per µL, mean (SD)	691 (295)	735 (286)	0.095
Current CD8 count per µL, mean (SD)	945 (431)	926 (456)	0.647
CD4 nadir <200 cells per µL, n (%)	71 (49.0)	241 (39.8)	0.044
Current cART use, n (%)	142 (97.9)	611 (98.9)	0.591
HIV RNA <50 copies/ml, n (%)	139 (94.6)	586 (95.6)	0.558
Time with HIV, years, mean (SD)	17.2 (9.6)	13.7 (8.8)	<0.001

SD, standard deviation; FEV_1_, forced expiratory volume in one second; FVC, forced vital capacity; LLN, lower limit of normal; AAT, Alpha-1 antitrypsin; cART, combination antiretroviral therapy.

### Plasma Concentrations of Inflammatory Markers

**Figure 1:**
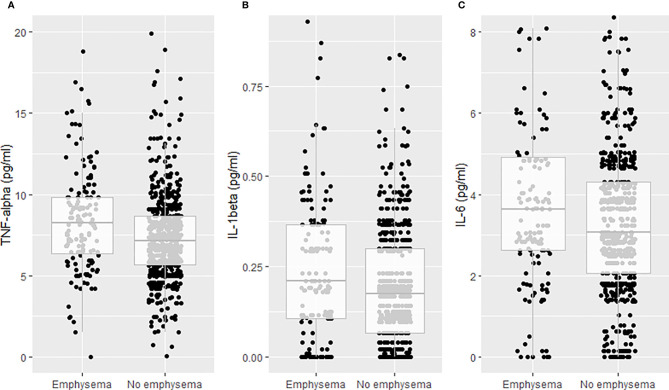
Plasma concentrations of tumor necrosis factor-alpha (TNFα), interleukin-1 beta (IL-1β) and interleukin-6 (IL-6) by radiographic emphysema. **(A)** TNFα. **(B)** IL-1β. **(C)** IL-6.

PLWH with emphysema had higher concentrations of TNFα (median (IQR): 8.2 (6.4-9.8) versus 7.1 (5.7-8.6) pg/ml, p<0.001), IL-1β (median (IQR): 0.21 (0.1-0.4) versus 0.17 (0.1-0.3) pg/ml, p=0.004) and IL-6 (median (IQR): 3.6 (2.6-4.9) versus 3.1 (2.0-4.3) pg/ml, p=0.023) than PLWH without emphysema (**Figure 1**). No differences in median cytokine concentrations were observed for IL-1RA, IL-2, IL-4, IL-10, IL-17A, IFNγ, sCD14 and sCD163 (**Table 2**). Also, AAT concentration and CD4/CD8 ratio < 0.4 did not differ between PLWH with and without emphysema.

**Table 2:** Cytokine and alpha-1 antitrypsin concentrations in PLWH with and without emphysema. Median values were calculated for cytokines with detection rates above 50%.

Cytokine	Emphysema(n=147)	No emphysema(n=618)	p-value
TNFα, median, pg/ml, (IQR)	8.2 [6.4-9.8]	7.1 [5.7-8.6]	<0.001
IL-1β, median, pg/ml, (IQR)	0.21 [0.1-0.4]	0.17 [0.1-0.3]	0.004
IL-1RA, median, pg/ml, (IQR)	43.6 [23.7-70.4]	42.6 [24.8-66.3]	0.594
IL-2, median, pg/ml, (IQR)	4.1 [0.0-7.0]	4.1 [0.0-7.1]	0.702
IL-6, median, pg/ml, (IQR)	3.6 [2.6-4.9]	3.1 [2.0-4.3]	0.023
IL-10, median, pg/ml, (IQR)	0.5 [0.4-0.9]	0.5 [0.4-0.8]	0.712
IFNγ, median, pg/ml, (IQR)	0.2 [0.0-1.7]	0.1 [0.0-1.5]	0.378
**Markers of monocyte activation**			
sCD14, median, ng/ml, (IQR)	3666 [3186-4299]	3597 [3031-4230]	0.158
sCD163, median, ng/ml, (IQR)	866 [662-1155]	852 [652-1.144]	0.651
**Biochemistry**			
AAT, g/L, (IQR)	1.4 [1.2-1.5]	1.4 [1.3-1.6]	0.305
CD4/CD8 ratio <0.4, n (%)	17 (11.6)	45 (7.5)	0.261

PLWH, people living with HIV; TNFα, Tumor necrosis factor-alpha; IL-1β, interleukin-1 beta; IL-1RA, interleukin-1 receptor antagonist; IL-2, interleukin-2; IL-6, interleukin-6; IL-10, interleukin-10; IFNγ, interferon-gamma; sCD14, soluble CD14; sCD163, soluble CD163; AAT, Alpha-1 antitrypsin.

### Elevated Cytokine Concentrations and Emphysema

Elevated TNFα (adjusted odds ratio (aOR): 1.89 [95%CI: 1.26-2.83], p=0.002) and IL-1β (aOR: 1.72 [95%CI: 1.14-2.59], p=0.009) were associated with emphysema in base models with adjustment for age and sex only (**Table 3**). These associations were robust in models adjusted for age, sex, ethnicity, smoking status, BMI and CD4 nadir; TNFα (aOR: 1.91 [95%CI: 1.23-2.95], p=0.004), IL-1β (aOR: 1.93 [95%CI: 1.25-3.00], p=0.003) (**Table 3**). Further, similar results were found when including cumulative smoking as a covariate in the logistic regression models, instead of smoking status. No associations between elevated concentrations of other inflammatory markers and emphysema were found (**Table 3**). Also, we found no association between a low CD4/CD8 ratio of <0.4 or 0.4-1.0 when compared to CD4/CD8 ratio > 1 and emphysema (aOR: 1.59 [95%CI: 0.76-3.34], p=0.216) and (aOR: 1.12 [95%CI: 0.71-1.76], p=0.625), respectively. In correlation analyses, we found evidence of a weak correlation between the two inflammatory markers significantly associated with emphysema, TNFα and IL-1β (rho=0.13, p<0.001). Both cytokines were therefore included in a final model, which yielded similar results: Elevated TNFα (aOR: 1.78 [95%CI: 1.14-2.76], p=0.011) and IL-1β (aOR: 1.81 [95%CI: 1.16-2.81], p=0.009).

**Table 3:** Multivariable logistic regression analyses for the association between elevated cytokine concentration (above the 75^th^ percentile) and emphysema in PLWH.

	Model 1^a^ Risk of emphysema High cytokine level *vs* low aOR [95% CI]	p-value	Model 2^b^ Risk of emphysema High cytokine level *vs* low aOR [95% CI]		p-value
_ _TNFα	1.89 [1.26-2.83]	0.002	1.91 [1.23-2.95]	0.004	
IL-1β	1.72 [1.14-2.59]	0.009	1.93 [1.25-3.00]	0.003	
IL-1RA	1.15 [0.75-1.76]	0.534	1.32 [0.83-2.11]	0.238	
IL-2	1.19 [0.78-1.81]	0.417	1.36 [0.87-2.13]	0.175	
IL-4	1.07 [0.67-1.70]	0.782	1.28 [0.79-2.09]	0.316	
IL-6	1.07 [0.70-1.66]	0.746	1.39 [0.87-2.23]	0.167	
IL-10	1.15 [0.75-1.75]	0.530	1.23 [0.78-1.92]	0.372	
IL-17A	1.27 [0.84-1.92]	0.262	1.23 [0.79-1.92]	0.352	
IFNγ	1.09 [0.71-1.67]	0.697	1.12 [0.71-1.76]	0.617	
sCD14	0.87 [0.56-1.36]	0.549	0.93 [0.58-1.48]	0.752	
sCD163	1.02 [0.66-1.58]	0.926	1.24 [0.78-1.98]	0.366	

^a^Model 1 adjusted for age and sex. ^b^Model 2 adjusted for age, sex, ethnicity, smoking status, BMI and CD4 nadir. Emphysema was defined as percentage of low attenuation area under 950 Hounsfield units (%LAA-950) > 5%. Abbreviations: PLWH, people living with HIV; TNFα, Tumor necrosis factor-alpha; IL-1β, interleukin-1 beta; IL-1RA, interleukin-1 receptor antagonist; IL-2, interleukin-2; IL-4, interleukin-4; IL-6, interleukin-6; IL-10, interleukin-10; IL-17A, interleukin-17A; IFNγ, interferon-gamma; sCD14, soluble CD14; sCD163, soluble CD163.

### Interaction Analyses

We found a statistically significant interaction between elevated IL-1β and smoking status on their effect on the odds for emphysema (p-interaction=0.020). Thus, the association between elevated IL-1β and emphysema was more pronounced in never-smokers (aOR: 4.53 [95%CI: 2.05-9.98], p<0.001) than in former (aOR: 1.29 [95%CI: 0.67-2.48], p=0.453) and current-smokers (aOR: 0.97 [95%CI: 0.37-2.54], p=0.950). In contrast, we found no evidence of statistical interaction between elevated TNFα and smoking status on their effect on the odds for emphysema (p-interaction=0.342).

### Sensitivity Analyses

Of 783 PLWH, 50 (6.4%) had visual emphysema according to the semi-quantitative assessment (visual score ≥2). Of these, 22 (44%) also had emphysema according to the primary outcome definition of %LAA-950 >5%. A Venn diagram showing the overlap between PLWH with emphysema according to the primary and the secondary outcome definition is available in the supplement (**Supplementary Figure 2**). In sensitivity analyses, using visual emphysema as the dependent outcome, elevated IL-1β was independently associated with emphysema in both minimal adjusted model (aOR: 2.51, [95%CI: 1.39-4.54], p=0.002) and the fully adjusted model including age, sex, ethnicity, smoking status, BMI category, CD4 nadir and TNFα (aOR: 2.62, [95%CI: 1.34-5.10], p=0.005). In contrast, the association between elevated TNFα and emphysema did not persist in sensitivity analyses (aOR: 1.05, [95%CI: 0.50-2.20], p=0.895). In explorative analyses, also using visual emphysema as the dependent outcome, we found an independent association between a low CD4/CD8 ratio <0.4 and emphysema (aOR: 3.71 [95%CI: 1.27-10.82], p=0.016).

## Discussion

In this large cross-sectional study, we included 783 well-treated PLWH without hepatitis B and C co-infection and explored associations between elevated concentrations of inflammatory markers and radiographic emphysema. PLWH with emphysema had higher concentrations of TNFα, IL-1β and IL-6 than PLWH without emphysema. Further, we found a strong association between elevated TNFα and IL-1β with emphysema in models adjusted for age and sex. These associations persisted in models adjusted for age, sex, ethnicity, smoking status, BMI category and CD4 nadir. These findings suggest that TNFα and IL-1β are independently associated with emphysema and may contribute to the pathogenesis of emphysema in PLWH. Moreover, in interaction analyses, we found the association between IL-1β and emphysema to be modified by smoking, with a more pronounced association in never-smokers, suggesting a pathogenesis independent of excessive smoking.

HIV-specific mechanisms for the development of emphysema, especially in the current cART era, are not fully understood (25). In the present study, we found an independent association between two markers of systemic inflammation, TNFα and IL-1β, and radiographic emphysema in PLWH. The main cellular source of both TNFα and IL-1β is activated mononuclear phagocytes such as macrophages, and the findings may thus reflect upregulation of inflammatory pathways in tissues, including the lungs. In this context, alveolar macrophages have been reported to harbor HIV, even among PLWH receiving suppressive cART, and may act as viral reservoirs (26). These HIV-infected macrophages can induce local inflammation and produce proteases in the lungs (27), suggesting there could be an HIV-specific mechanism for the destruction of lung parenchyma and development of emphysema. Thus, the observed association between elevated peripheral TNFα and IL-1β with emphysema, may be a consequence of a spill-over of cytokines from the affected lung tissue, where the cytokines are most active, and into the systemic circulation (28). However, experimental studies have shown that elevated cytokine levels measured in plasma correlate poorly with elevated cytokine levels in the lungs (29).

Alternatively, the association between elevated TNFα and IL-1β and emphysema could reflect an upregulation of systemic inflammation which, in turn, could enhance inflammation in the lungs and accelerate the destruction of lung parenchyma and lead to emphysema in PLWH (9). This hypothesis is supported by a large study investigating the burden of and risk factors for emphysema in 1446 PLWH on cART. The study demonstrated that besides smoking, peripheral leukocytosis and elevated CRP were the most important risk factors for emphysema in PLWH on cART (11). Indeed, persistent inflammation and immune activation in PLWH have been reported repeatedly, even in those receiving suppressive cART, and inflammation is now considered a hallmark of HIV infection (12, 30). We previously found several pro-inflammatory markers, including TNFα and IL-6, to be higher in PLWH than in uninfected controls (20). Moreover, persistent inflammation has consistently been linked to the development of non-AIDS comorbidities other than pulmonary diseases, in particular cardiovascular and metabolic diseases (14, 15). Mechanisms contributing to comorbidity are thought to include residual viral replication due to HIV reservoirs, co-infections, disruption of gut microbiota leading to microbial translocation of antigens and cumulative ART toxicity (31). Of note, gut-associated microbial translocation could lead to the activation of macrophages and subsequent increased release of circulating pro-inflammatory markers, such as IL-1β and TNFα, as observed in the present study.

Interestingly, we found a significant interaction between IL-1β and smoking on their effect on the odds for emphysema, with a more pronounced effect seen in never-smokers. Thus, it is likely that systemic inflammation may contribute to the pathogenesis of emphysema, even in PLWH without exposure to tobacco. In this context, it is noteworthy that emphysema originates around the small airways and that never-smoking PLWH have been found to have small airway dysfunction (32). Moreover, a recent study that included 159 never-smoking PLWH on cART and 75 never-smoking uninfected controls, found a 4-fold increased risk of CT signs of emphysema in PLWH compared to uninfected controls (7). Taken together, these findings suggest that HIV itself, possibly through upregulation of IL-1β dependent inflammatory pathways, could cause destruction of lung parenchyma compatible with emphysematous changes. However, the cross-sectional design of the present study prevails us from drawing conclusions on causality.

The relationship between inflammatory markers and radiographic emphysema in PLWH has only been investigated in two previous studies (6, 19). Both of these studies were conducted in the Examinations of HIV Associated Lung Emphysema (EXHALE) Study, and in one of these studies, the main finding was an association between elevated sCD14 and radiographic emphysema in PLWH (6). However, 34% had detectable viral replication and the prevalence of injection drug use and marijuana use was high (32% and 85%, respectively) and not adjusted for, which may explain the discrepancies with the results of the present study. Moreover, in the second study, the authors failed to demonstrate a significant association between elevated sCD14 and emphysema (19), questioning the role for sCD14 in emphysema in PLWH. In the same study, an independent association between a low peripheral CD4/CD8 ratio (<0.4) and emphysema was found, suggesting that residual immune activation may contribute to the pathogenesis of emphysema in PLWH. In the present study, we did not find an association between a low CD4/CD8 ratio and radiographic emphysema. However, comparison of the two studies is embedded by the use of different methods for quantification of radiographic emphysema. The quantitative assessment of emphysema by %LAA-950 > 5% used in the present study most likely represents both emphysema plus trace emphysema, whereas visual assessment, which was used in the EXHALE study, may reflect later stages of emphysema (21, 33). Indeed, in explorative analyses using visual emphysema as the dependent outcome, we did find an independent association between a low CD4/CD8 ratio (<0.4) and emphysema, suggesting that residual immune activation may be involved in the pathogenesis of more severe emphysema rather than the mild.

We did not find an association between elevated IL-6 and emphysema, although IL-6 concentrations were found to be higher in PLWH with emphysema compared to PLWH without emphysema in crude analyses. This is in line with a large study conducted in the general population (n=1928), where no association between circulating IL-6 and emphysema was found (10). In contrast to the findings of the present study, the authors did not find evidence of an association between elevated TNFα and emphysema. Thus, one could speculate that PLWH may experience different phenotypes of emphysema than uninfected controls, as a consequence of pathological mechanisms mediated by up-regulation of inflammatory markers such as TNFα and IL-1β which could explain the higher prevalence of respiratory symptoms in PLWH compared to uninfected controls, previously found in our and other cohorts (8, 34). Of note, IL-1β was the only cytokine associated with visual emphysema, suggesting that IL-1β may be a more clinically relevant biomarker than TNFα. As such, we found the overlap between the quantitative and the visual assessment of emphysema to be low, a finding that has also been reported in previous studies (21, 35). The low correlation between the two distinct methods for emphysema assessment used in our study could also be explained by the fact that the cut-off for the quantitative assessment (%LAA-950) was set at 5% to allow a wide range of emphysematous changes to be included, and the cut-off for visual emphysema was set at ≥ 10%. Thus, the visual definition may reflect more severe and clinically relevant stages of emphysema in our study. Finally, the findings of the current study emphasize the need for paying attention to and including never-smokers in the risk group for emphysema in PLWH along with persons with a history of tobacco exposure.

Our study has strengths and limitations. First, the cross-sectional nature of our study does not allow us to draw conclusions on causality. Furthermore, we are unable to determine whether the observed association between elevated plasma concentrations of TNFα and IL-1β and radiographic emphysema in PLWH is due to local inflammation in the lung or rather reflects a more general state of chronic systemic inflammation. Thus, measuring the concentration of the inflammatory markers locally in the lungs would have provided further mechanistic insight. Finally, we did not include uninfected controls and therefore cannot conclude whether the observed associations are unique to PLWH. The strengths of the study include a large sample size, a quantitatively and highly reproducible method for assessment of emphysema (33) and adjustments for potential confounders.

In conclusion, we found an independent relationship between TNFα and IL-1β and radiographic emphysema in PLWH, suggesting that systemic inflammation may be involved in the pathogenesis of emphysema. The association between IL-1β and emphysema was more pronounced in never-smokers, emphasizing the need for PLWH without a history of smoking to be evaluated for symptoms and risk factors for emphysema, as well as PLWH with tobacco exposure. Whether elevated markers of inflammation are only associated with the prevalence of emphysema or it could predict development or progression of emphysema in PLWH remains unknown, and future studies should reveal whether elevated markers of systemic inflammation could serve as a biomarker to predict development of HIV-associated emphysema.

## Data Availability Statement

The datasets presented in this article are not readily available due to restrictions from the Danish Data Protection Agency. Requests to access the datasets should be directed to Susanne Dam Nielsen, sdn@dadlnet.dk.

## Ethics Statement

The studies involving human participants were reviewed and approved by The Committee on Health Research Ethics of the Capital Region of Denmark. The patients/participants provided their written informed consent to participate in this study.

## Author Contributions

RT was responsible for concept, data collection, statistical analysis, and drafted the manuscript. AR was responsible for concept, data collection, and edited the manuscript. HH and MT contributed to cytokine analysis and edited the manuscript. AM provided input for statistical analyses and edited the manuscript. HR, TB, JG, AB, and JV were responsible for concept and edited the manuscript. SO and SN were responsible for concept, data collection and have had content review and editing input. All authors contributed to the article and approved the submitted version.

## Funding

This work was supported by Rigshospitalet Research Council and The Novo Nordisk Foundation. JV is supported by the NIHR Manchester BRC. AB was supported by The Lundbeck Foundation during the conduct of this study (Grant R219-2016-762).

## Conflict of Interest

RT: reports a grant from Rigshospitalet Research Council. TB: reports grants from Pfizer, grants from Novo Nordisk Foundation, grants from Lundbeck Foundation, grants from Simonsen Foundation, grants and personal fees from GSK, grants and personal fees from Pfizer, personal fees from Boehringer Ingelheim, grants and personal fees from Gilead, personal fees from MSD, grants from Lundbeck Foundation, grants from Kai Hansen Foundation, outside the submitted work. AM: has received travel support, honoraria and consultancy fees from ViiV, Gilead and Eiland and Bonnin PC. JV: reports honoraria from consulting/presenting from AstraZeneca, Boehringer-Ingelheim, Chiesi, GSK, Novartis and Sanofi and a grant from Boehringer-Ingelheim, outside the submitted work. SN: unrestricted research grants from Novo Nordisk Foundation and Rigshospitalet Research Council. Advisory board activity for Gilead and GSK/ViiV.

The remaining authors declare that the research was conducted in the absence of any commercial or financial relationships that could be construed as a potential conflict of interest.

## Abbreviations

AAT, alpha-1 antitrypsin; BMI, body mass index; cART, combination antiretroviral therapy; COCOMO, Copenhagen Comorbidity in HIV Infection Study; EXHALE, Examinations of HIV Associated Lung Emphysema Study; CT, computed tomography; HIV, human immunodeficiency virus; HU, Hounsfield unit; IFNγ, interferon-gamma; IL-1β, interleukin-1 beta; IL-1RA, interleukin-1 receptor antagonist; IL-2, interleukin-2; IL-4, interleukin-4; IL-6, interleukin-6; IL-10, interleukin-10; IL-17A, interleukin-17A; sCD14, soluble CD14; sCD163, soluble CD163; TNFα, tumor necrosis factor-alpha; PLWH, people living with HIV; %LAA-950, low attenuation area under −950.
