# Supplementary figures and images for: Independent Associations of Tumor Necrosis Factor-Alpha and Interleukin-1 Beta With Radiographic Emphysema in People Living With HIV
Source: Front Immunol. 2021 Apr 14;12:668113. doi: 10.3389/fimmu.2021.668113 (PMC8080065; doi:10.3389/fimmu.2021.668113)

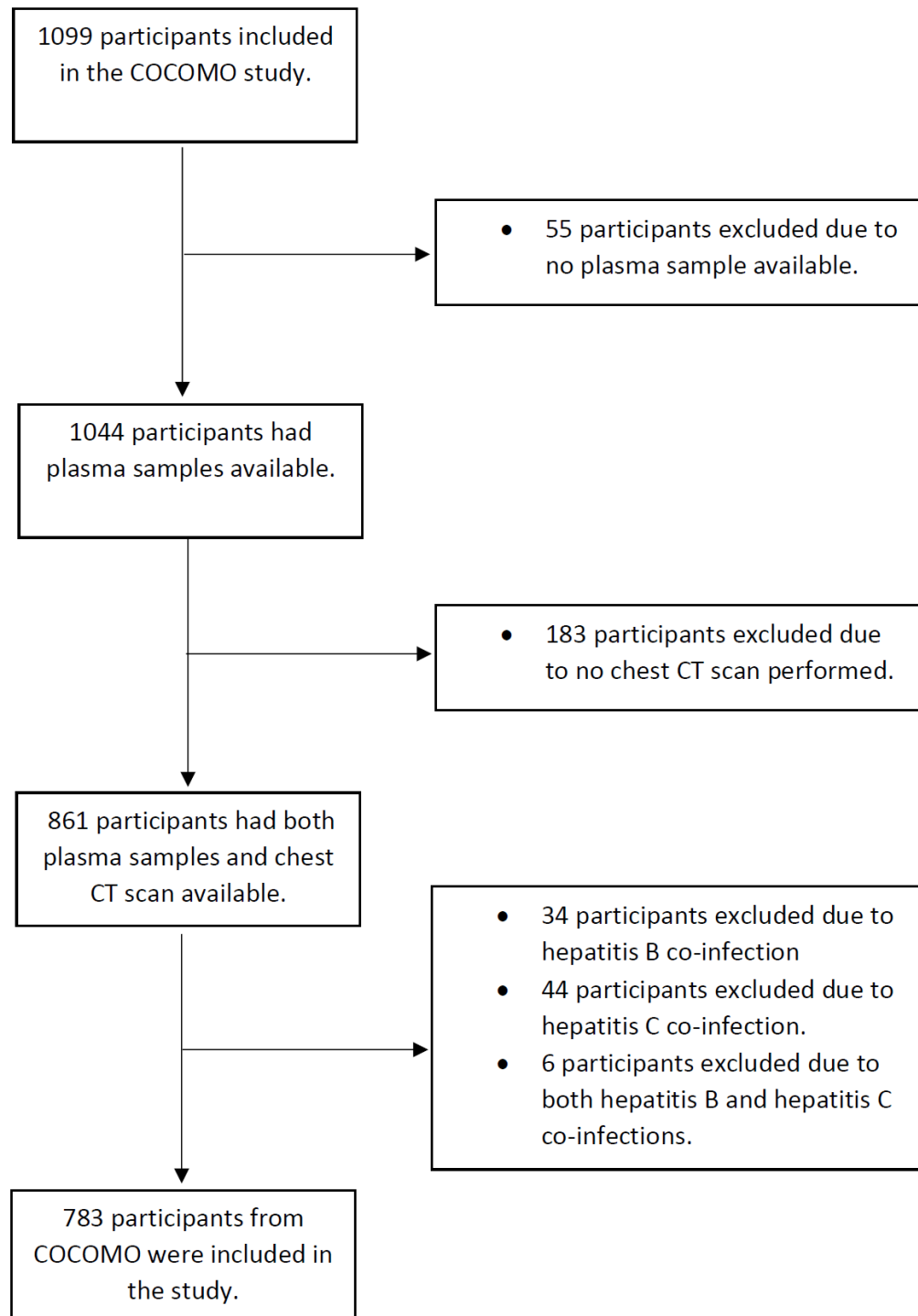

**Supplementary Figure 1:** Study participant flowchart.

Supplement: Supplementary file 1 [file Image_1.pdf]
