# Supplementary material for: Independent Associations of Tumor Necrosis Factor-Alpha and Interleukin-1 Beta With Radiographic Emphysema in People Living With HIV
Source: Front Immunol. 2021 Apr 14;12:668113. doi: 10.3389/fimmu.2021.668113 (PMC8080065; doi:10.3389/fimmu.2021.668113)
Supplement: Supplementary file 2 [file Image_2.pdf]

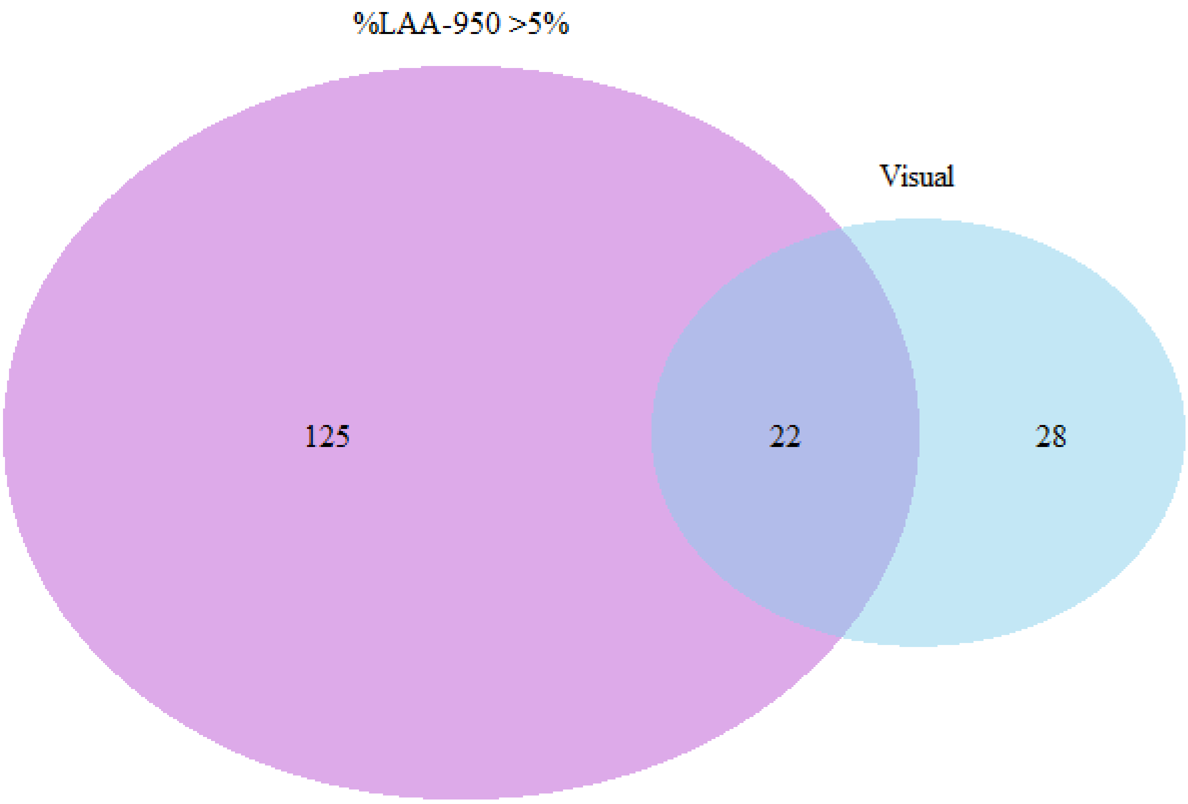

Supplementary Figure 2: Venn diagram showing the overlap of PLWH with emphysema according to the %LAA-950 >5% definition of emphysema and the visual definition of emphysema (visual score ≥2).
